# Manganese Encephalopathy Caused by Homemade Methcathinone (Ephedrone) Prevalence in Poland

**DOI:** 10.3390/nu13103496

**Published:** 2021-10-03

**Authors:** Bogusław Habrat, Andrzej Silczuk, Anna Klimkiewicz

**Affiliations:** 1 Department of Prevention and Treatment of Addictions, Institute of Psychiatry and Neurology, 02-957 Warsaw, Poland; habratb@hotmail.com (B.H.); silczuk@wp.pl (A.S.); 2 Department of Public Health, Medical University of Warsaw, 02-091 Warsaw, Poland; 3 Department of Psychiatry, Medical University of Warsaw, 02-091 Warsaw, Poland

**Keywords:** prevalence, manganese encephalopathy, methcathinone, ephedrone, manganism

## Abstract

Manganese encephalopathy is a known disorder in occupational medicine. A serious phenomenon has been the emergence of manganese encephalopathy in intravenous users of homemade methcathinone (ephedrone). A short survey was developed for clinical environments dealing with people who use psychoactive substances. The data were obtained from 72 rehabilitation therapy centers. Surveys carried out in about a third of Polish centers dealing with providing medical assistance to people addicted to substances other than alcohol and tobacco have shown that over 4% of people treated there had symptoms of manganese encephalopathy, of which more than half are people in whom the probability of a clinical diagnosis of this disorder is significant. It has been shown that knowledge of manganese encephalopathy is none or minimal in more than 70% of the surveyed institutions. An urgent need for personnel training in this field was pointed out. Attention was paid to the importance of disseminating good review articles on new and dynamically developing problem phenomena.

## 1. Introduction

The symptoms of manganese encephalopathy in healthy physical workers professionally exposed to manganese generally may appear after years or decades of continuous, chronic exposure. Manganese (Mn) accumulates in the brain regions with dopaminergic pathways (mainly the basal ganglia, most in the pale globe), potentially leading to dopaminergic dysfunction. Symptoms for a clinical diagnosis of manganese encephalopathy can assemble in different configurations (see Box 1). 

**Figure 1:**
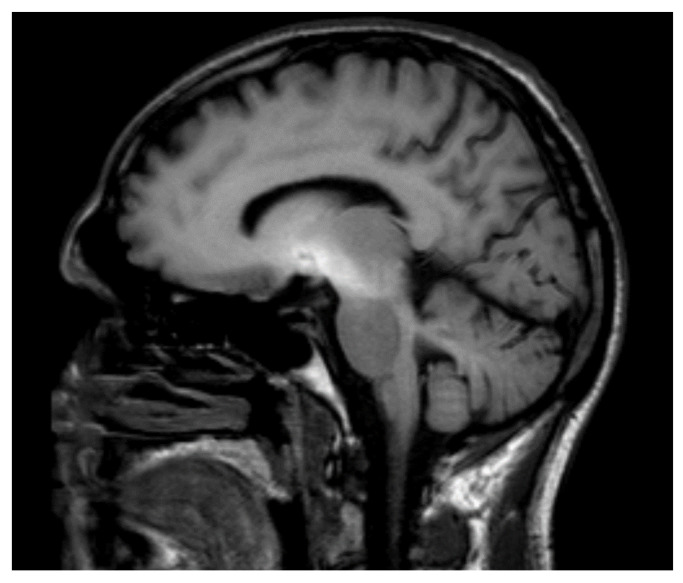
MRI T1 basal ganglia in intravenous methcathinone users. Nonenhanced, T1 weighted MR images show markedly hyperintense signals in the medial and lateral part of the globus pallidus, putamen, and in a lesser extend in part of thalamic nuclei.

MRI imaging for manganese encephalopathy is typically the discovery of a symmetrical intensification of a dependent T1 signal, mainly located in the basal ganglia, especially in the pale knobs and cerebri branches (see Figure 1). Changes in the hypothalamus and midbrain are less commonly described [1,2,3,4]. The course of encephalopathy is unfavorable. Symptoms are usually permanent; symptomatic treatment (mainly anti-Parkinson drugs) is usually ineffective. Despite confirmation of the elimination of Mn from the brain (neuroimaging) and blood (serum levels) the insensitivity of symptoms of manganese encephalopathy remains unchanged [5,6,7].

Box 1 **Symptoms of the manganese encephalopathy.**

- Body posture
  - sometimes leading to backward falls,
- Propulsion and/or retropulsion,
- Characteristic dystonic disturbances in the facial muscles
  - a forced smile grimace (reminiscent of "risus sardonicus"),
- Dysarthria (speech significantly reduced to anarthria in advanced cases, monotonous speech) [8],
- Extrapyramidal symptoms, such as:
  - muscle stiffness,
  - slowdown,
  - impaired movement,
  - impaired facial expressions,
  - difficulties in precise movements, mainly of the hands,
  - micrograph (writing in smaller and smaller letters),
- Pseudobulbar symptoms: inadequate, forced laughter or crying,
- The so-called. "Rooster gait" (stepping on toes) and compensatory protrusion of the chest [9,10],

Cognitive deficits [11,12].

Manganese encephalopathy is a disorder often found in patients in occupational medicine. It also occurs in industrial workers chronically exposed (for decades) to manganese compounds. Manganese compounds, including manganese fume, have a current Federal OSHA PEL of 5 milligrams per cubic meter of air (5 mg/m^3^) [13]. According to the Agency for Toxic Substances and Disease Registry (ATSDR) toxicological profiles, the normal ranges of Mn levels in body fluids are 4–15 μg/L in blood, 1–8 μg/L in urine, and 0.4–0.85 μg/L in serum. Manganese encephalopathy may occur in welders [14], steelworkers [15], metal ore miners [16], manual workers producing batteries [17], and persons that have contact with certain insecticides. Due to the dominant extrapyramidal symptoms in the clinical image, it is often called manganese-induced parkinsonism (MIP). Less commonly, manganese encephalopathy can be found in people with chronic liver disease and electrolyte balance impairment [18,19], in people with acute intoxication with Mn compounds [20], and some cases of chronic parenteral nutrition [21]. Neurotoxic effects of manganese on fetuses have also been reported [22] and found in children exposed to environmental manganese poisoning [23,24]. The main contributing factor leading to MIP in children is that the placenta provides some protection against unwanted chemical exposures during fetal development, but it is not an effective barrier to environmental pollution. Additionally, the blood–brain barrier, which protects the adult brain from many toxic chemicals, does not fully form until about six months after birth. At much lower doses than those that affect adult brain functions, exposure to the manganese during early fetal or early childhood development can cause brain injury [25]. That leads to the fact that the manganese neurodevelopmental toxicity has recently become a major public health concern. High levels of Mn exposure, as confirmed by elevated Mn hair levels, have been linked to hyperactivity and oppositional behaviors in children [26,27,28]. Other reports also demonstrate that decreased intellectual functions [29] and Attention-Deficit Hyperactivity Disorder [30] among children correlate with high concentrations of heavy metals in local drinking water. Because the majority of Mn is bound to erythrocytes, even minor hemolysis results in increased serum levels [31].

A relatively new and surprising phenomenon was the emergence of manganese encephalopathy in intravenous users of homemade methcathinone (ephedrone). Most of those subjects were presenting MIP symptoms. The first cases were described in Russia in the early 1990s [32] and later in the decade [33,34]. They were called ephedrone encephalopathy (the common name for methcathinone), although it was established relatively early that the neurotoxic agent is not methcathinone, but manganese [35]. Observations and studies were published mainly in Russian-language publications and magazines with limited circulation. Soon, there were reports of single cases and small groups of intravenous users of manganese-based methcathinone preparations, mainly from post-Soviet countries: Georgia [8,36], Latvia [37,38], Estonia [39,40], and neighboring countries, e.g., Turkey [41,42] where manganese methcathinone was called a “Russian cocktail.” Papers mainly dealt with the description of neurological symptoms, brain imaging studies, treatment trials, and short-term catamnesis. Soon, reports from Western countries were presented, mainly case studies, and this phenomenon concerned mainly emigrants from post-Soviet countries [43,44,45]. Relatively little knowledge about manganese encephalopathy and most of the numerous case reports were from neuroradiologists and neurologists who focused mainly on the little-known but typical image in neuroimaging studies (mainly MRI) [46,47,48,49]. The first cases of encephalopathy caused by manganese used to produce methcathinone in Poland were observed in 2011–2012 [50].

The technology of homemade production of methcathinone (ephedrone) involves the chemical reaction of the pseudoephedrine contained in some complex “anti-cold” drugs with potassium permanganate in an acidic environment (acetic acid, citric acid) [51]. The methcathinone thus obtained is injected intravenously without chemical purification of the preparation from manganese compounds. Manganese is a compound whose strong neurotoxic effects are relatively well known in experimental animal models [52,53] and people chronically (most often for decades) exposed to manganese compounds [54]. Only one study analyzed the effect of preparations containing methcathinone and manganese compounds on the brains of apes [55].

In Poland, the production of methcathinone from drugs containing pseudoephedrine has been known since the late 1990s [56]. Homemade chemical preparation and possession of methcathinone is forbidden by current Polish law. Initially the information on the production of methcathinone was transmitted orally and then via online forums. In 2007, it was experimentally confirmed that the synthesis described on the Internet leads to methcathinone [57]. However, the presence of manganese in the chemical process thus obtained was not of interest. Although homemade methcathinone production is simple and cheap, and semi-finished products are readily available, it appears that the phenomenon of intravenous use of manganese with methcathinone was limited in scope until 2010. Until that time no evidence of encephalopathy was seen in subjects dependent on homemade ephedrone (methcathinone). There is a probability that it was caused by the progressive westernization of drug addiction and access to cheap street drugs (designer drugs, also proscribed by Polish law), which were an attractive alternative to the homemade methcathinone. Since 2011, severe cases of manganese encephalopathy started to be diagnosed at the Institute of Psychiatry and Neurology in Warsaw in intravenous users of various psychoactive substances, including users of homemade manganese methcathinone [58]. The problem was identified fairly quickly. Over 30 patients were diagnosed, and treatment attempts were made. At the same time, the Institute of Psychiatry and Neurology in Warsaw launched a training program on diagnosing manganese encephalopathy. Some trainings addressed to professionals as well as meetings with patients were conducted. During the training sessions, the rehabilitation therapists revealed that numerous cases of probable manganese encephalopathy were found in the past, which were not recognized or treated.

In this article, the results of a quick survey among rehabilitation centers are presented. Besides some basic demographic items, we asked and focused on three main questions: how widespread is the phenomenon of intravenous use of methcathinone with manganese in Poland, what are the dynamics of this phenomenon, and what is the level of knowledge among therapists about the symptoms of manganese encephalopathy?

## 2. Materials and Methods

A paper–pencil survey was developed for clinical environments dealing with people who use psychoactive substances. All questionnaires (see the Appendix A.) were sent by mail with self-addressed envelopes. The survey, cover letter, and educational materials were directed to the heads of all registered Polish public rehabilitation centers. The surveys consisted of two main parts.

The first part was to determine the nature of the center: stationary, detoxification, stationary rehabilitation, outpatient, and others. Additionally, questions were asked about the number of drug users in treatment in the last 3 years.

The second part contained questions about substantive issues: the number or percentage of intravenous users of methcathinone (ephedrone) preparations from pseudoephedrine containing medicines (Sudafed^®^, Acatar^®^, etc.) after chemical reaction with potassium permanganate; the approximate number of patients in the past five years whose clinical image was very similar to the description given in the cover letter to this survey and attached review article [5]; the number of patients in past five years who had from 1 to 3 symptoms listed in the clinical description; the number of patients who had these symptoms before 2010. 

Furthermore, the variants of diagnostic and therapeutic procedures in patients with neurological concerns were studied and a comparison of the frequency of using intravenous ephedrone preparations with manganese before and after 2010 was made. Finally, the value of the attached training materials was evaluated and the center’s employees’ knowledge about manganese encephalopathy was assessed.

The materials sent to the heads of centers included the cover letter with the invitation for participation in the study, explaining the genesis and purpose of the research, and briefly describing the clinical picture of manganese encephalopathy. In addition, it contained the survey described above and a copy of the review article on manganese encephalopathy [5], and an addressed and franked envelope to send back filled questionnaires. 

The centers were selected based on a list of addiction treatment centers in which over 30 people addicted to substances other than alcohol and tobacco are treated annually (data collected from the Department of Public Health of Poland of the Institute of Psychiatry and Neurology in Warsaw). Voluntary participation in research and anonymity was promised, and most centers sealed their surveys or envelopes.

Research results and other numerical values were collected in an electronic version in MS Office Excel. Simple statistical measures were performed with the main focus being on frequencies and frequency distribution.

## 3. Results

The results of this study may constitute a single message. The presence of methcathinone users in Poland is confirmed. The secondary outcome of this article reveals the importance of education and information on novel phenomena. These are the main findings of this research.

### 3.1. Questionnaire

Out of 129 questionnaires sent (10 returned because the recipient was not found), 72 questionnaires were sent back, which constitutes 55.8% of the questionnaires. Four centers reported that they do not deal with the medical aspects of substance use anymore.

In this study, data were obtained from 72 rehabilitation therapy centers, including 21 stationary rehabilitation units, 2 detoxification units, 36 outpatient clinics, 3 combining inpatient and outpatient treatment, and from 10 centers outside of these categories. In total, in these centers, 15,819 people were treated for drug dependence.

### 3.2. Intravenous Methcathinone Use and Manganese Encephalopathy Symptoms

Information about 462 people who have been receiving methcathinone–manganese preparations intravenously in the last six years was obtained. At least 370 cases whose clinical picture corresponded to manganese encephalopathy were identified in the surveyed centers. Another 289 clients had three or fewer symptoms. This allows an approximate extrapolation of the number of all cases by doubling the reported people with suspected encephalopathy (surveys were sent back by more than half of the facilities in Poland). Thus, it can be expected that the number of people requiring in-depth neurological diagnostics can be close to 1000 cases.

Of the total number of 659 subjects with neurological symptoms occurring in manganese encephalopathy identified in the responding rehabilitation centers, only 58 people (8.8%) had these symptoms before 2010, i.e., the time when legal regulations were introduced significantly limiting access to legally sold “designer drugs”. This could suggest that the introduction of these restrictive provisions could have resulted in reaching for home-produced methcathinone: simply and cheaply made from generally available products. For assessment of the direct impact of the presentation of legal act penalizing the designer drugs on methcathinone use, most respondents (52 centers—72.2%) chose the option: it is difficult to say, and from only eight centers (11.1%) offered an opinion that the phenomenon of intravenous methcathinone and manganese use intensified after the penalization of “designer drugs”, which is more or less the same as the statement that it occurs at a similar level before and after 2010 (9 centers—12.5%). Only one center (1.4%) considered that the “designer drugs” policy on counteracting drug addiction had fulfilled its task and reduced the number of users of methcathinone with manganese. Two centers (2.8%) stated that they were not in operation long enough to assess this phenomenon.

### 3.3. Professional Knowledge of Manganese Encephalopathy

The survey shows that knowledge of the clinical picture of manganese encephalopathy is not common. Of the studied 72 centers:

- 13 centers (18%) assessed their staff’s knowledge of manganese encephalopathy as none;
- 11 of them (15.3%) declared the need for training;
- 2 did not see such a need due to the currently “non-medical nature” of their center;
- 38 centers (52.8%) declared minimal knowledge in this area;
- 21 centers (29.2%) declared sufficient competence in dealing with people with suspected manganese encephalopathy;
- of which 15 (20.1%) considered that they could still be further trained.

According to surveys results, 70.8% of centers assessed their knowledge about manganese encephalopathy as none or minimal. The willingness to train in the clinical aspects of manganese encephalopathy was expressed in 64 out of 72 centers (88.9%).

The brief information materials and a copy of the review paper on manganese encephalopathy in manganese-contaminated users of methcathinone were very positively assessed. Only 3 centers (4.2%) declared that the attached materials did not increase their knowledge and do not affect clinical practice in these centers. The remaining 69 centers (95.8%) emphasized the value of the attached materials, which increased diagnostic alertness and improved the management of persons suspected of manganese encephalopathy.

## 4. Discussion

The starting point of the research was numerous signals from practitioners that there are drug-dependent patients presenting symptoms previously unseen in this group. Their syndrome was also unknown to most consulting neurologists. The aim of the study was not to present a clinical description of the patients with manganese encephalopathy. The authors focused mainly on an initial assessment of manganese encephalopathy prevalence and an initial assessment of clinical issues (diagnosis and treatment) knowledge. The secondary aim was to provide the basic knowledge (attached ME review) to the drug rehabilitation units and centers. The authors have assumed that any form of a complicated clinical trial would not be methodologically correctly possible in the presented research model. The questionnaires were anonymous, mainly so that the centers would not be afraid of being identified and reassessed. Self-addressed envelopes were attached to the questionnaires mailing. All activities undertaken in this study aimed to maximize the response rate to gain as much information as possible. The preliminary clinical trials conducted so far were based on trials on small (*n* < 20) groups. Clinical verification of more than 1000 possible cases of manganese encephalopathy in intravenous users of methcathinone is impossible for organizational and financial reasons.

Table 1 summarizes the first, preliminary epidemiological studies with more than ten cases. They show the extent of the phenomenon used in manganese encephalopathy and intravenous users of homemade methcathinone contaminated with manganese. So far, they provide some insight, but do not entirely depict the scale of the phenomenon, as only clinical studies in which the size of the group is known was mentioned. The results of the presented research cannot be added up and analyzed because studies from one center often concerned the same people. There are only general statements about the “epidemic” and “large number of young addicts” in Estonia [38]. “The Parkinsonism epidemic recently reported in Russia, Ukraine, Estonia, and other parts of Eastern Europe” is also mentioned [59].

The total number of people who received intravenous methcathinone with manganese was greater than the number of likely cases of manganese encephalopathy but less than the total number of probable cases of manganese encephalopathy and individuals with single (up to three) symptoms suggesting the possibility of this diagnosis. In many centers that reported this phenomenon, more cases of encephalopathy were reported than for intravenous users of methcathinone tainted with manganese. It cannot be ruled out that the question about the use of homemade methcathinone with manganese was not included in the routine set of questions in the interview about the use of psychoactive substances. 

Admittedly, it is reported that the use of certain stimulants increases the risk of Parkinson’s syndrome [11,60], this phenomenon does not seem to occur more commonly in the group of Polish patients (usually these syndromes develop longer and are less severe).

**Figure 2:**
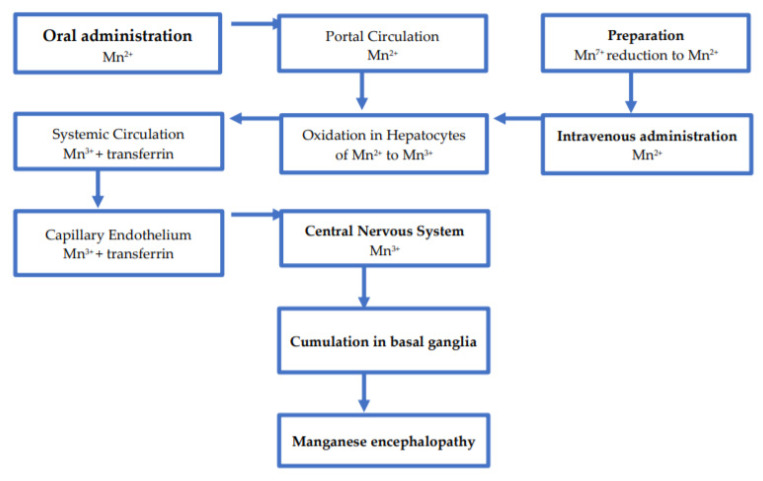
Hypothetical models of Manganese blood–brain transport.

Some hypotheses have been raised on the Mn blood–brain barrier crossing by orally ingesting Mn-containing agents [69]. It may be assumed that Mn from intravenous administration of methcathinone partially shares the metabolic routes as presents Figure 2.

Potassium permanganate (MnVII) is a strong oxidizing agent that is widely used in the chemical industry and laboratories. Homemade production of methcathinone, whose production uses the oxidation of pseudoephedrine with the use of potassium permanganate as an oxidant allows the obtaining of the end product, unfortunately contaminated with manganese (MnII) ions. Intravenous administration of this substance introduces ions (MnII) into the bloodstream, which in the further stages of metabolic processes (MnIII) combine with transferrin through the blood–brain barrier reaching the basal ganglia. For example, their presence in the pale globe (see Figure 1) leads to symptoms of so far unknown etiology that are surprising clinicians.

## 5. Limitations of the Study

It may be difficult to assess to what extent the centers that sent the data could be representative for all facilities. Moreover, the study is limited by the fact that the belief about knowledge was examined, and not the actual state of knowledge of the providers of therapeutic services in the studied centers. Demographical data concerning reported patients are limited due to the designed method of this study. The detailed studies, e.g., manganese levels, NMR, neurological consultations, and clinical descriptions of patients were not performed to correlate various addiction parameters. The authors need to underline that the design of this study was not clinical. The survey was short and straightforward in order not to discourage responding centers from sending the answers back.

## 6. Conclusions

Surveys carried out in about one of three of centers dealing with providing medical assistance to people addicted to substances other than alcohol and tobacco have shown that over 4% of people treated there had symptoms of manganese encephalopathy, of which more than half are people in whom the probability of clinical diagnosis of this disorder is significant. It has been shown that knowledge of manganese encephalopathy is absent or minimal in more than 70% of the surveyed institutions. That means that patients suffering from manganese encephalopathy remain undiagnosed and left without intervention or prevention even though for several decades, both clinical image and cellular effect and blood–brain barrier passage of Mn mechanisms have been described [2,5,6,31,32,44,69]. An urgent need for personnel training in this field is indicated, which is supported by published reviews so far [12]. Attention is drawn, and supported by these research results, to the importance of disseminating good and educational review articles on these new and dynamically developing problem phenomena. It seems justified to design in-depth studies as well as a follow-up study to provide more information concerning the treatment provided to diagnosed patients, the diagnostic methods, and undertaken intervention. The study may constitute the basis for obtaining funding for conducting large clinical trials in Poland and Europe.

## Figures and Tables

**Table 1:** The number of studied groups from studies on manganese encephalopathy.

Authors	Year	Country	N	M:F	Average Age
Sikk et al. [61]	2011	Estonia	20	17:3	-
Sikk et al. [62]	2013	Estonia	38	31:7	33
Sikk et al. [62]	2013	Estonia	24	-	-
Khatiashvili et al. [36]	2007	Georgia	10	-	-
Chinchaladze et al. [63]	2012	Georgia	22	-	-
Giorgishvili et al. [64]	2016	Georgia	3	-	-
Bonnet et al. [65]	2014	Georgia	28	27:1	-
Rusz et al. [8]	2014	Georgia	28	-	39.9 +/−4.9
Stepens et al. [37]	2010	Latvia	10	8:2	40 (30–55)
Stepens et al. [38]	2011	Latvia	23	-	-
Stepens et al. [7]	2014	Latvia	18	17:1	36.5 (23–47)
Golasik et al. [48]	2014	Poland	24	17:7	
Levin [35]	2005	Russia	21	14:7	-
Ismailova et al. [66]	2005	Russia	65	60:5	-
Fedorova et al. [33]	2007	Russia	65	-	-
Levin & Datieva [34]	2013	Russia	35	-	-
Sanotsky et al. [58]	2007	Ukraine	13	13:0	-
Selighova et al. [67]	2008	Ukraine	13	13:0	-
Djamshidian et al. [68]	2012	Ukraine	15	-	-

## Data Availability

Dataset is with authors. Available on request.

## Appendix A. The Questionnaire on the Prevalence of Manganese Encephalopathy among Patients of Addiction Treatment Centers

Please fill the below questionnaire:

1. Center:
  1. Inpatient.
    1. Detoxifying.
    2. Rehabilitation.
    3. Other (what kind? …).
  2. Outpatient.
  3. Inpatient and outpatient.
  4. Other (what kind? …).
2. In the last six years approx. … patients addicted to substances (other than alcohol) were treated in our center (please provide the number of patients)
3. Of them, about … (please provide the number of patients) used intravenous ephedrone preparations from medicines containing pseudoephedrine (Sudafed, Acatar, etc.) - prepared with potassium permanganate.
4. In the last six years we had approx. … (please provide the number) patients whose clinical picture was very similar to the description of manganese encephalopathy that was given in the cover letter of this questionnaire and the review papers.
5. In the last six years we had approx. … (please provide the number) patients who developed single (up to 3) symptoms listed in the clinical description as above.
6. About … (please provide the number) of patients had had above mentioned symptoms also before 2010′.
7. The management of patients with suspected manganese encephalopathy (please mark appropriate):
  - The suspicions were quickly verified by neurologist who performed MRI of the
    brain, as well as measured blood manganese levels.
  - The patients were referred to neurologists who did not verify the suspicion of the
    manganese encephalopathy or did not provide recommendation for the treatment.
  - Patients were referred to other medical centers.
  - We were not aware of the possibility of any further diagnosis of patients.
  - We were unable to refer patients for further examinations due to:
    lack of patients’ insurance.
    refusal from other medical centers.
    other (please specify)…
8. The phenomenon of using ephedrone and the manganese preparations (please mark appropriate):
  1. Lasts for many years, also before 2010′ and has not undergone major changes.
  2. Intensified after the introduction of the legal changes in 2010.
  3. The legal changes introduced in 2010 had reduced the use of this preparation.
  4. It’s hard to say.
9. What is the level of knowledge about the manganese encephalopathy in medical staff of your center?
  1. None and they do not need training.
  2. None and they do need training.
  3. Minimal but and they do not need training.
  4. Minimal and they do need training.
  5. Sufficient and they do not need training.
  6. Sufficient, but training can increase their competence.
  7. Excellent and they do not need training.
10. Attached information about manganese encephalopathy:
  1. Significantly and sufficiently increased the alertness of the staff to this disorder.
  2. Did not change our treatment policy because the staff is familiar with clinical symptoms of manganese encephalopathy.
  3. Is unnecessary.
